# Deep learning for identifying corneal diseases from ocular surface slit-lamp photographs

**DOI:** 10.1038/s41598-020-75027-3

**Published:** 2020-10-20

**Authors:** Hao Gu, Youwen Guo, Lei Gu, Anji Wei, Shirong Xie, Zhengqiang Ye, Jianjiang Xu, Xingtao Zhou, Yi Lu, Xiaoqing Liu, Jiaxu Hong

**Affiliations:** 1 Department of Ophthalmology, The Affiliated Hospital of Guizhou Medical University, Guiyang, China; 2 Hisense Medical, Qingdao, China; 3 Boston Children Hospital, Harvard Medical School, Boston, USA; 4 Department of Ophthalmology and Visual Science, Eye, and ENT Hospital, Shanghai Medical College, Fudan University, 83 Fenyang Road, Shanghai, China; 5 Shanghai Key Laboratory of Visual Impairment and Restoration, Science and Technology Commission of Shanghai Municipality, Shanghai, China; 6 Key Laboratory of Myopia, Ministry of Health, Shanghai, China; 7 Deepwise AI Lab, 21st Floor, China Sinosteel Plaza, No.8, Haidian Ave, Beijing, China

**Keywords:** Diseases, Medical research

## Abstract

To demonstrate the identification of corneal diseases using a novel deep learning algorithm. A novel hierarchical deep learning network, which is composed of a family of multi-task multi-label learning classifiers representing different levels of eye diseases derived from a predefined hierarchical eye disease taxonomy was designed. Next, we proposed a multi-level eye disease-guided loss function to learn the fine-grained variability of eye diseases features. The proposed algorithm was trained end-to-end directly using 5,325 ocular surface images from a retrospective dataset. Finally, the algorithm’s performance was tested against 10 ophthalmologists in a prospective clinic-based dataset with 510 outpatients newly enrolled with diseases of infectious keratitis, non-infectious keratitis, corneal dystrophy or degeneration, and corneal neoplasm. The area under the ROC curve of the algorithm for each corneal disease type was over 0.910 and in general it had sensitivity and specificity similar to or better than the average values of all ophthalmologists. Confusion matrices revealed similarities in misclassification between human experts and the algorithm. In addition, our algorithm outperformed over all four previous reported methods in identified corneal diseases. The proposed algorithm may be useful for computer-assisted corneal disease diagnosis.

## Introduction

Corneal disease is a major cause of reversible blindness worldwide, ranking second only to cataracts^1^, with an estimated 6.8 million people in India^2^ and 3.2 million in China^3^ having corneal blindness in at least one eye. Importantly, vision loss due to corneal disease may be avoidable through early diagnosis and appropriate therapy^4^. The assessment of the ocular surface, primarily the cornea and conjunctiva, by ocular slit-lamp examination is the foundation of corneal disease diagnosis. However, this is highly dependent on the grader’s clinical experience, which is time-consuming and may have interobserver variation on the same patient. Automated grading of medical images could be used to address these issues by reducing the physicians’ workload, increasing the efficiency and reproducibility of screening programs, and improving patient prognosis through early detection and treatment.

Recent advances on deep learning algorithms, in particular convolutional neural networks (CNN), have made it possible to learn the most predictive features of disease directly from medical images when given a large dataset of labeled examples^5,6^. Esteva et al.^7^ proposed a dermatologist level classification of skin cancer via fine-tuning a pre-trained Inception-v3^8^ network. Chilamkurthy et al.^9^ conducted a retrospective study to detect critical findings in CT scans of head via deep learning algorithms. In the field of eye diseases identification, recent studies have shown the ability to identify retinal and optic nerve diseases via retinal photographs^10–13^ or optical coherence tomography (OCT) images^14,15^. Gulshan et al.^10^ demonstrated the detection of diabetic retinopathy through fine-tuning a pre-trained Inception-v3^8^ network on retinal fundus images. Gargeya et al.^11^ performed automated identification of diabetic retinopathy using a ResNet-based architecture^16^. Similarly, Li et al.^12^ adopted a Inception-v3^8^ network for glaucomatous optic neuropathy detection using color fundus images while Burlina et al.^13^ applied both a pre-trained model and a newly trained from scratch model for automated grading of age-related macular degeneration from color fundus images. In contrast, Schlegl et al.^14^ and Treder et al.^15^ both proposed automated detection of macular diseases using OCT images.

However, to date, there have been few studies on diagnosing ocular surface diseases. Long et al.^17^ developed a technique for diagnosis of congenital cataracts with acceptable diagnostic accuracy. However, their method was trained based on images covering the pupil area only. By using corneal confocal microscopy images, Williams et al.^18^ employed a convolutional neural network with data augmentation to develop an algorithm for analyzing the corneal sub-basal nerve plexus in patients with diabetic neuropathy and their method showed excellent performance for the quantification of corneal nerve biomarkers. Unlike Long’s^17^ and Williams’s work^18^, to cover a wider spectrum of ocular surface diseases, we utilized the whole ocular surface image and were not limited to the pupil. This makes our algorithm capable of detecting corneal diseases related to the peripheral cornea and limbus.

In this study, we sought to develop an effective deep learning algorithm for multiple corneal disease identification by processing ocular surface images. Then, we performed an evaluation of the algorithm’s diagnostic performance on outpatients in a prospective manner.

## Methods

In the current study, our dataset comes from two major eye centers in China: the Shanghai Eye, Ear, Nose, and Throat Hospital and the Affiliated Hospital of Guizhou Medical University, Guizhou Province. From April 2017 to October 2017, we retrospectively collected 5,325 ocular surface slit-lamp images including 870 from normal subjects and 4,455 from patients with one of the five tested eye diseases for developing the deep learning algorithm (Supplemental Fig. 1). For the prospective study, we obtained patient’s informed consent to apply our algorithm for screening a separate clinic-based dataset with 510 images from these two major eye centers from June 2018 to July 2018. All ocular surface slit-lamp images were obtained by the IM 900 or IM 600 digital slit lamp photography system (Haag-Streit, Switzerland). Only images covering and centering around the cornea were used from patients. Pictures from normal subjects were randomly selected from the database, while pictures from patients were consecutively collected during the study period. The institutional review board of Shanghai Eye, Ear, Nose and Throat Hospital approved this project (EENTIRB20170607), and we conducted the research according to the tenets of the Declaration of Helsinki.

### Ocular surface disease photograph grading and reference standard

**Figure 1:**
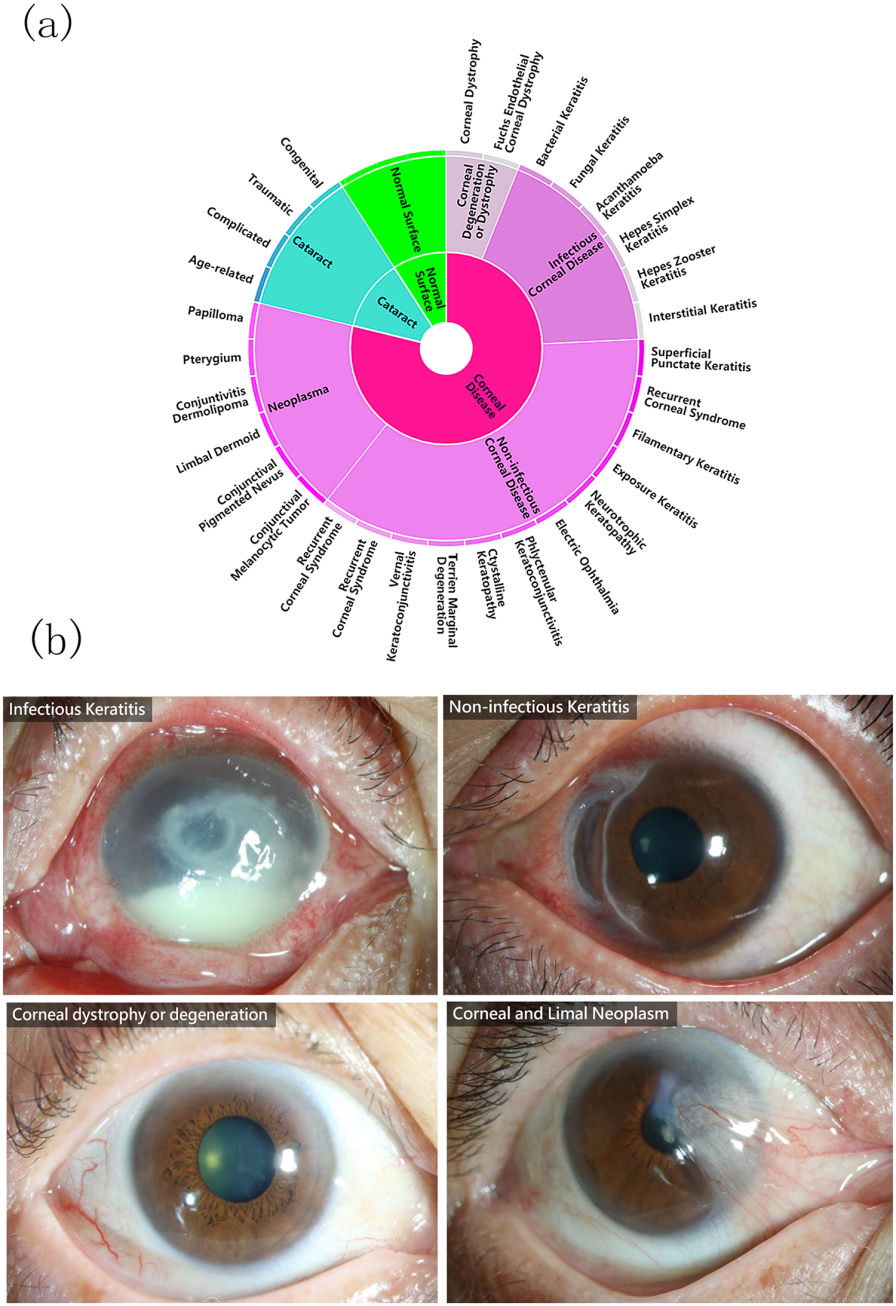
A schematic illustration of the novel taxonomy and example test set images. (**a**) A subset of the top two levels of the taxonomy of ocular surface diseases affecting the corneal area. (**b**) Example images from four representative corneal diseases including infectious keratitis, non-infectious keratitis, corneal dystrophy and degeneration, and corneal neoplasm.

Thirty-two ophthalmologists were invited to grade the images of the retrospective database. During the training of ophthalmologists, a dataset of 90 images (30 infectious keratitis, 10 non-infectious keratitis, 20 corneal dystrophy or degeneration, and 30 corneal neoplasm) was used for the test. The participants’ results were compared with those of two senior corneal specialists (H.G. and J.H.), and those participants did not complete the training until they achieved a *κ* value of 0.75 or more (A *κ* value of 0 indicates that observed agreement is the same as that expected by chance, and a κ-value of 1 indicates perfect agreement. A κ-value of 0.6 to 0.8 indicates substantial agreement and 0.8 to 1.0 almost perfect agreement). As a result, only 20 ophthalmologists qualified as graders to classify the images. Each photograph was reviewed with the same standard and graded via face-to-face communication between two ophthalmologists. All 5325 ocular surface slit-lamp images had personal medical history, etiology test, and the original diagnosis recorded in the medical charts, graders were asked to review and check all information, and then classify the images, as shown in Fig. 1. Corneal disease was defined as any disease affecting the corneal area, including infectious keratitis, non-infectious keratitis, corneal dystrophy or degeneration, and corneal and limbal neoplasm. Finally, 5325 ocular surface slit-lamp images were collected over a 7-month period (Retrospective dataset, Table 1) for the training and validation phases of our study. The mean number of images graded per ophthalmologist was 637 (range 556–746).

**Table 1:** Proportion of ocular surface slit-lamp images covering the cornea area in the training, validation, and test datasets.

	Retrospective dataset n (%)	Prospective dataset n (%)
Normal Subjects	870 (16.3%)	87 (17.1%)
Cataract	1,860 (34.9%)	160 (31.4%)
Infectious keratitis	845 (15.9%)	86 (16.9%)
Non-infectious keratitis	785 (14.7%)	81 (15.9%)
Corneal dystrophy or degeneration	550 (10.3%)	54 (10.6%)
Corneal Neoplasm	415 (7.8%)	42 (8.2%)
Total	5,325	510

*In the current study, corneal diseases include infectious keratitis, non-infectious keratitis, corneal dystrophy or degeneration, and ocular surface neoplasm affecting the cornea.

### Development and validation of the deep learning algorithm in a retrospective dataset

To take the advantage of fine-grained information embedded within the images, a domain taxonomy structure has been defined to partition diseases into coarse-to-fine classes hierarchically arranged in a Pie structure as shown in Fig. 1. Inspired by Esteva et al.^7^, the taxonomy was derived by ophthalmologists using a bottom-up procedure: individual diseases, initialized as leaf nodes, were merged based on clinical and visual similarity, until the entire structure was connected. This aspect of the taxonomy is useful in generating training classes that are both well-suited for machine learning classifiers and medically relevant. The first two levels of the taxonomy are used in performance validation. However, extension to more levels can be easily implemented via our flexible and extensive framework.

In the current study, 5325 high-quality ocular surface images were collected for the training framework and the validation. Among the 5325 fully gradable photographs, 20% of them (i.e., 772 images) were selected randomly using a random sampling and treated as the testing dataset, and the remaining images were used as the training set. It needs to be mentioned that the disease distributions (i.e., the ratio of each disease in each subset) in the training and validation sets are the same.

### The proposed hierarchical deep learning framework

**Figure 2:**
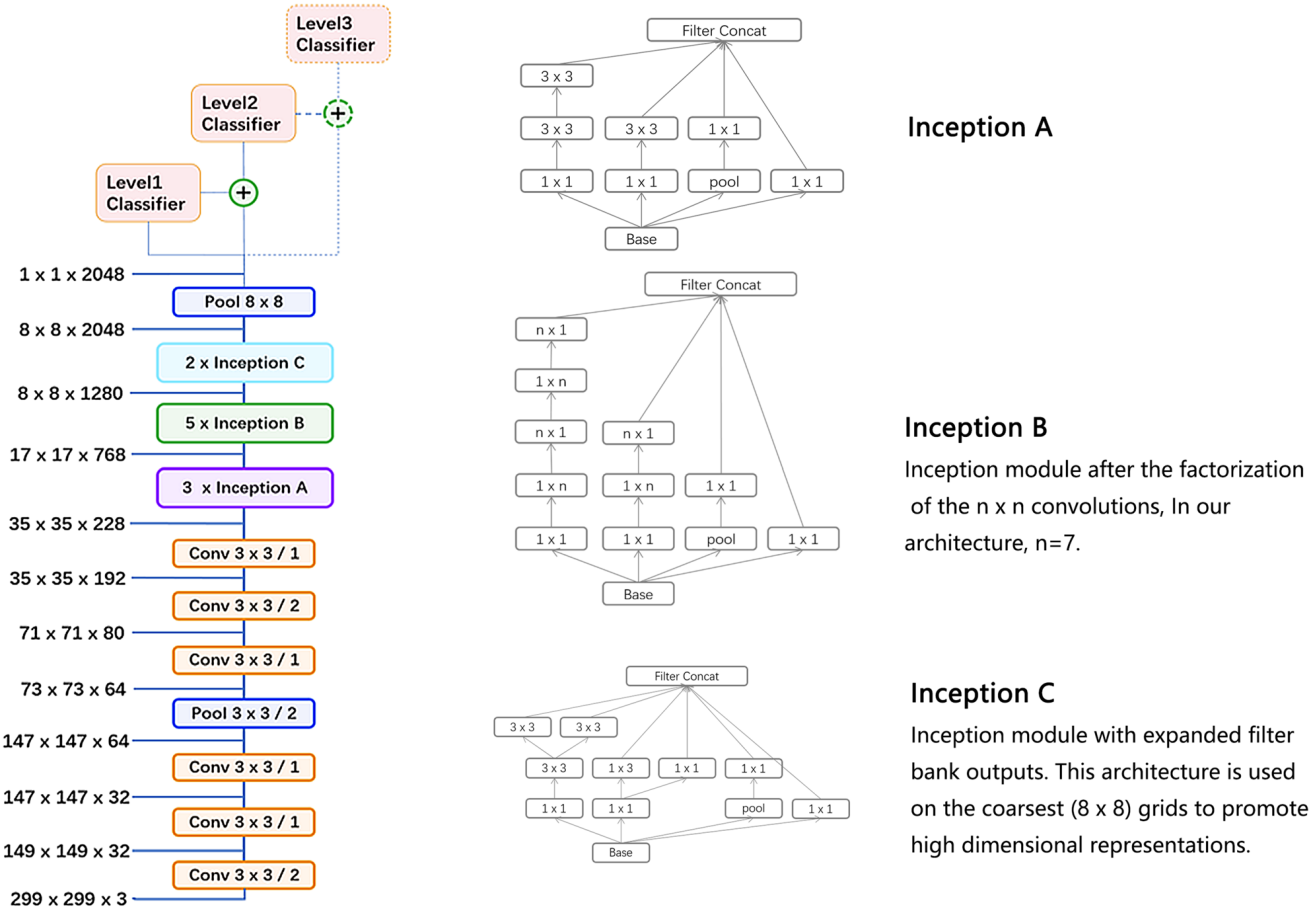
The proposed network architecture based on the backbone network of Inception v3. In our framework, a family of multi-task multi-label classification layers were utilized hierarchically representing various levels of eye disease. Next to each module, identify the size of input and output. ‘Conv 3 × 3/2’ indicates that a 3 × 3 convolution kernel is used and stride = 2. Different spatial factorized inception modules are presented here. Inception A contains the factorization of the original 5 × 5 convolutions; Inception B factorizes general nxn convolutions, and Inception C has expanded the filter bank outputs.

As shown in Fig. 2, the proposed hierarchical deep learning framework is a flexible and extensible hierarchical learning system that is composed of a family of multi-task multi-label learning classifiers representing different levels of eye disease classification derived from the hierarchical eye disease taxonomy. We utilized an Inception-v3 convolutional neural network architecture^7^ as the backbone of the proposed framework and the final classification layer of the Inception-v3 network was replaced with our novel multi-task multi-label classification layers, hierarchically representing various levels of eye disease classification. As a result, the classification results of each lower level classifier can be used as the prior for corresponding higher levels of classifiers, thereby improving the final classification performance.

We train the model by minimizing our novel multi-level eye disease-guided loss function consisting of multiple levels of losses. The objective function for two levels can be represented as follows: $Loss=\alpha *leve{l}_{{1}_{loss}}+\left(1-\alpha \right)*level\_2\_loss$

Where Loss is the total loss of the final model, level_1_loss and level_2_loss represent the loss of first level and second level of eye diseases identification, respectively (level 1 and level 2 disease labels were listed in Fig. 1a). $\alpha$ is a weight parameter which is used to control the balance between the two losses. Since multiple diseases may simultaneously coexist, we use sigmoid function for each class instead of the commonly used softmax function which is normally used in the case of one choice from all classes. For the loss function of each level, we applied Kaiming He’s focal loss^19^, which not only reduces the impact of data imbalance, but also is better than the usual loss function for model training since focal loss down weight easy classified samples and puts training focus on hard negatives. The focal loss function can be represented as:

$$FL\left({p}_{t}\right)={{-(1-p}_{t})}^{\gamma }\mathrm{log}({p}_{t})$$

where

$$ p_{t}  = \left\{ {\begin{array}{ll}    p & {if\;y = 1}  \\    {1 - p} & {otherwise}  \\   \end{array} } \right. $$

${{(1-p}_{t})}^{\gamma }$ is a modulating factor to the cross-entropy loss, with tunable focusing parameter $\gamma \ge 0$.

The whole network was trained via fine-tuning the parameters pre-trained on the ImageNet dataset^20^ (approximately 1.28 million images of 1000 object categories) across all layers with our dataset. Due to the unbalanced property of data, various of data augmentation methods (such as flipping, color jitter, etc.) were also applied for all classes independently to balance the data. Since we use a multi-task multi-label structure, each task branch consists of several stacked fully connected (FC) units. First, the multi-task branches were trained by freezing the backbone’s weights for 5 epochs. We used Adam optimizer and used the learning rate of 0.0001 and epsilon of 0.1. During this process, the classification loss weight for level 1 classifier and level 2 classifier was 3:7. Then, we performed a multi-step re-training strategy. In this strategy, we gradually unfroze the layer weights in steps, with the first few layers being unfrozen last. In these steps, we used progressively reduced learning rates (0.0001, 0.00001, and so on with other parameters unchanged). Every step lasted 20 epochs. We used Facebook’s Pytorch^21^ deep learning framework with strong GPU acceleration to train, validate, and test the algorithm networks.

**Figure 3:**
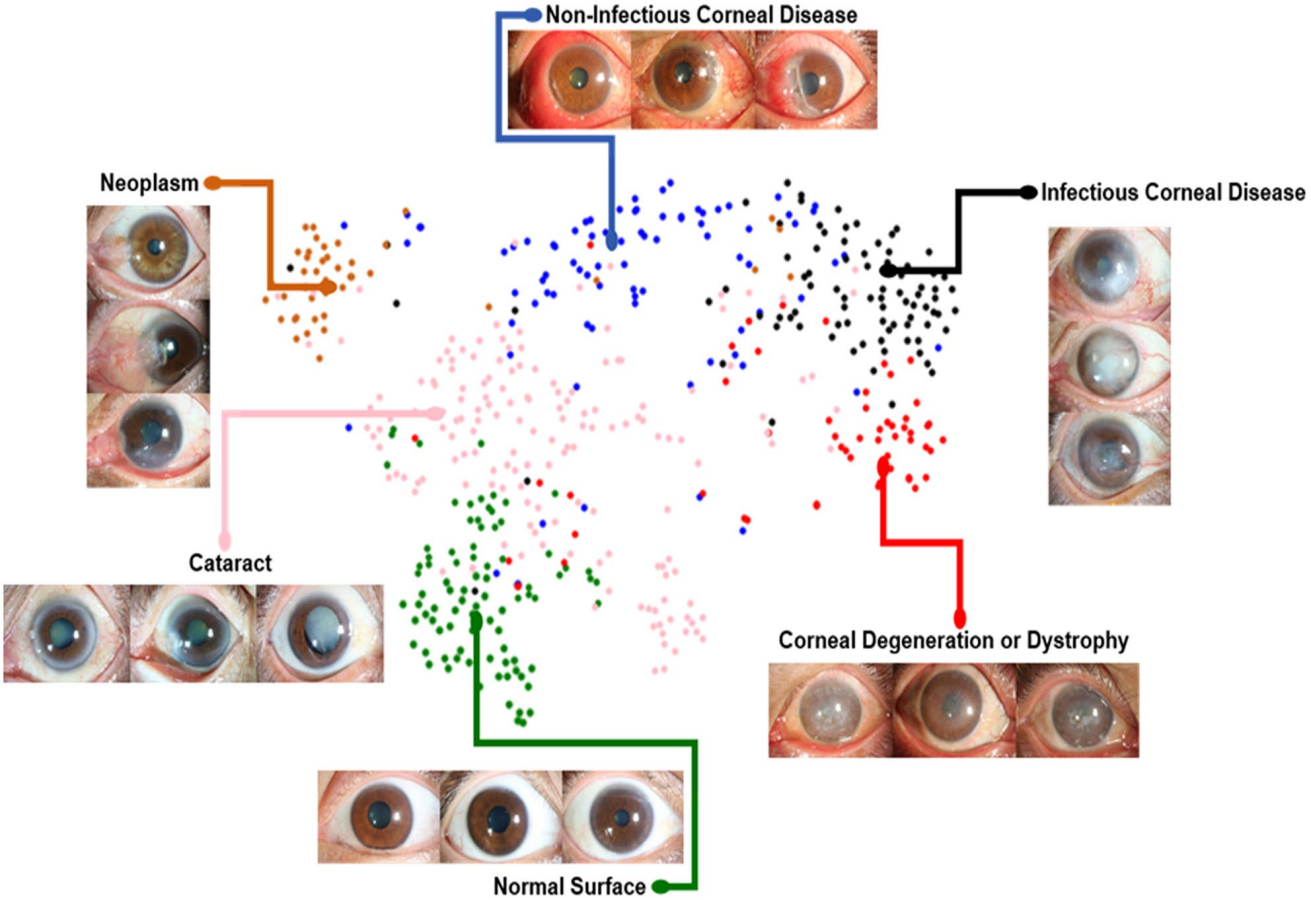
The t-SNE visualization of the last hidden layer representations in the algorithm for diseases from the prospective dataset (510 images). Colored point clouds represent disease categories, showing how the algorithm clusters the diseases. Clusters of points represent our method’s ability to objectively separate normal patients from those with corneal diseases. Each point represents an ocular surface image projected from the 2048-dimensional output of the last hidden layer of the Inception-v3 backbone into two dimensions. We see clusters of points of the same clinical diseases. Patients with cataracts cluster in the center, while normal cornea cluster on the lower left. Corneal and limbal neoplasm cluster on the upper left. Among corneal disease, infectious keratitis is split across the corneal disease point cloud, indicating that it is prone to confusion with non-infectious keratitis and corneal dystrophy, which is in agreement with the confusion matrices results (Fig. 4).

**Figure 4:**
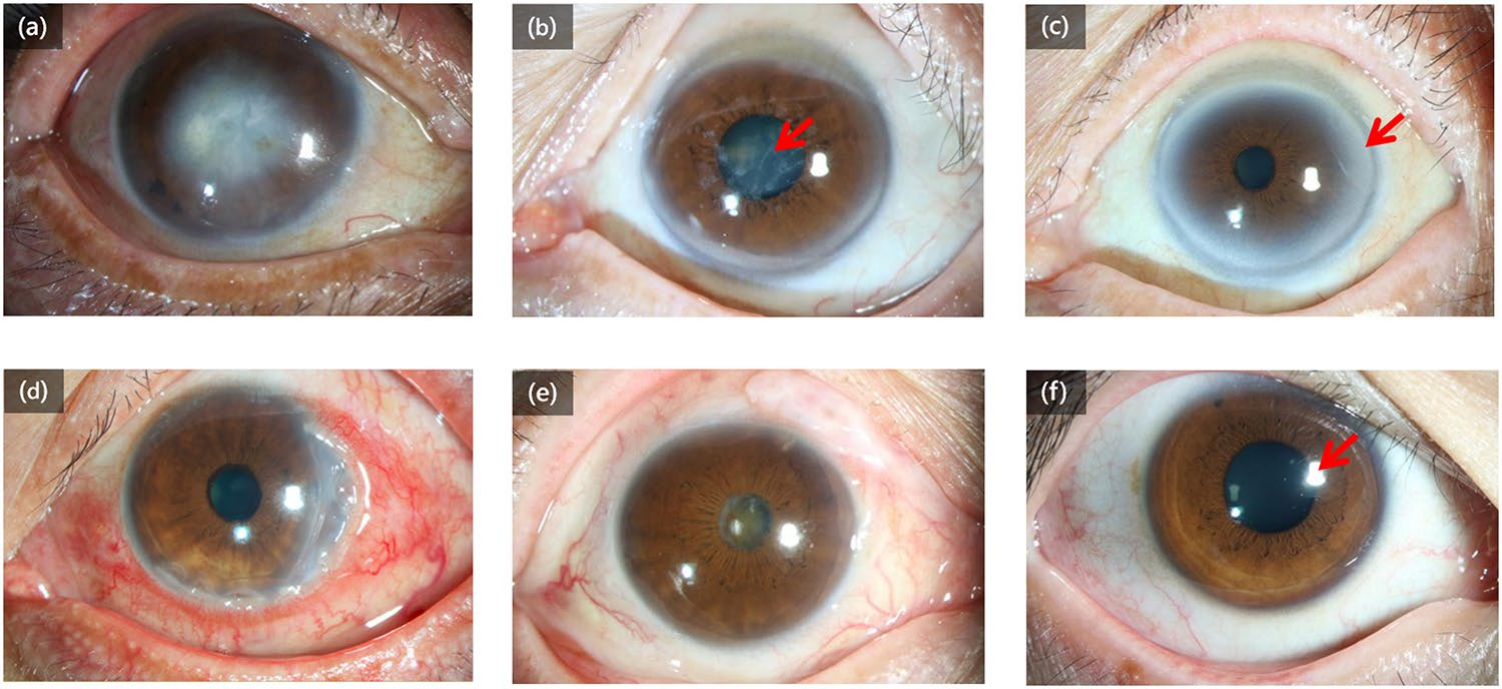
Reasons for misclassification output from the algorithm in the prospective dataset. (**a**) A patient with central corneal lesions was diagnosed as cataracts by the algorithm. (**b**) A patient with corneal dystrophy was diagnosed as cataracts by the algorithm. The misclassification in these two images was primarily the result of the corneal lesion being limited to the pupil area (red arrow). (**c)** A patient with arcus senilis (red arrow), a common corneal degeneration in older subjects, was diagnosed as cataracts by the algorithm. (**d**) A patient with non-infectious keratitis was diagnosed as infectious keratitis by the algorithm and the junior ophthalmologist. (**e**) A patient with a complicated case of cataracts was diagnosed as infectious keratitis by the algorithm. (**f)** A normal subject was diagnosed as infectious keratitis by the algorithm. We postulate that multiple and irregular corneal reflecting light (red arrow) may manifest as a corneal lesion to the algorithm.

We also examined the internal features via t-distributed Stochastic Neighbor Embedding (t-SNE)^22^ where point clouds with different colors represent different disease categories. As demonstrated in Fig. 3, each point represents an eye image projected from the n-dimensional output of the last hidden layer of Inception-v3^8^ backbone into two dimensions. We see clusters of points from the same clinical classes. This visualization represents our method’s ability to objectively separate normal patients from those cases for referral. As shown in Fig. 3, patients with cataracts cluster in the center, while normal cornea cluster on the lower left. Corneal and limbal neoplasm cluster on the upper left. Among corneal disease, infectious keratitis is split across the corneal disease point cloud, indicating that it is prone to confusion with non-infectious keratitis and corneal dystrophy (Fig. 3, 4).

### Prospective study of the deep learning algorithm in a clinical setting

Two tertiary eye centers (one in Shanghai City from East China and one in Guiyang City from West China) were involved in the algorithm’s validation. 1,218 outpatients were invited to participate in this study, of which 510 agreed to receive the test and took ocular surface slit-lamp photos before their physician visits. Informed consent was obtained from all subjects. A software practitioner participating in this study fed the images as inputs to the trained deep learning software model. The algorithm generates a probability distribution over the classification nodes in a sequential top-down manner, i.e., one level by one level. For each case, a particular disease was diagnosed if the probability value of that corneal disease subtype was ranked highest by the algorithm. ROC curves were plotted. To compare our algorithm’s sensitivity and specificity to that of 10 ophthalmologists on the diagnostic task of these 510 cases, each ophthalmologist was asked the diagnosis of the images.

### Confusion matrices

Figure 4 shows the confusion matrix of our method over the identified five diseases and normal case of the validation strategy in comparison to the tested ophthalmologists. This demonstrates the misclassification similarity between the algorithm and human experts. Element (i, j) of each confusion matrix represents the empirical probability of predicting class j given that the ground truth was class i.

### Statistical analysis

The area under the ROC curve (AUC) with 95% confidence intervals was used to evaluate the algorithm’s diagnostic performance. In addition, the system’s accuracy, sensitivity, and specificity were also evaluated.

## Results

**Figure 5:**
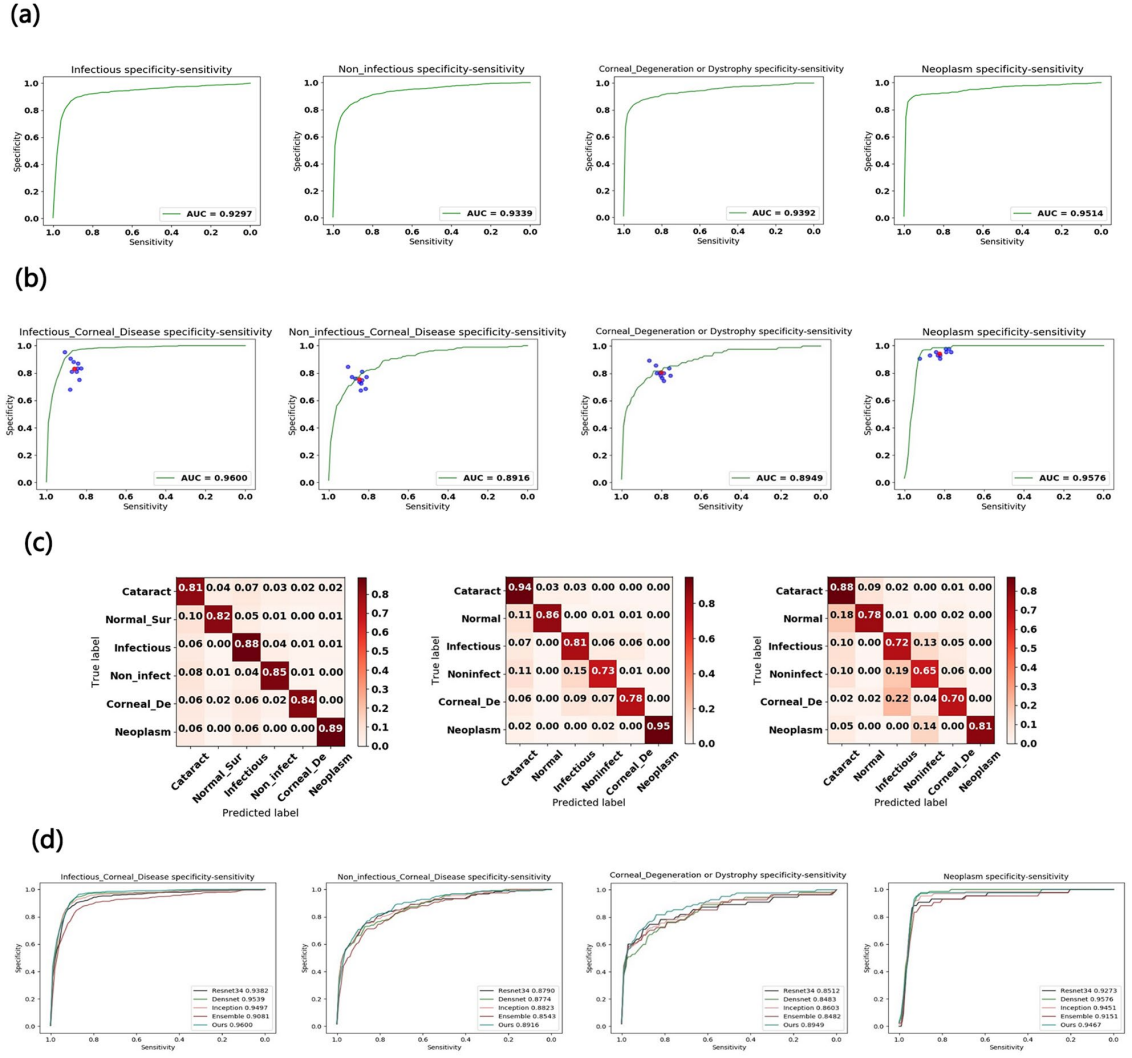
Corneal diseases identification performance of the proposed deep learning algorithm and ophthalmologists. (**a**) The algorithm achieves acceptable AUC values in identifying corneal diseases on the testing dataset with 772 images. (**b**) Our algorithm was tested against 10 ophthalmologists for the 510-subject dataset. The algorithm outperforms the average of the 10 ophthalmologists at corneal inflammation disease (infectious and non-infectious keratitis) and achieves performance on par with them in corneal dystrophy, corneal degeneration, and neoplasm when using ocular surface photographic images. (**c**) Confusion matrices for diagnosis of normal, cataract and four common corneal diseases between the algorithm and two ophthalmologists with varying levels of clinical experience reveal similarities in misclassification between human experts and the algorithm. The distribution across column 1—cataract—is pronounced in all plots, demonstrating that many lesions are easily confused with this disease. Note that both the algorithm and the ophthalmologists noticeably confuse infectious and non-infectious keratitis (diseases 2 and 3) with each other, with ophthalmologists erring on the side of predicting infectious keratitis. The distribution across row 5 in all plots shows the difficulty of classifying corneal dystrophy or degeneration, which tends to be diagnosed as infectious keratitis. (**d**) Performance comparison with four existing methods, namely Resnet34, Densenet, Inception-v3, and Ensemble. Our algorithm achieved better AUC than Resnet34, Densenet, Inception-v3, and Ensemble in most of corneal diseases. Only Densenet has a higher AUC than ours in diagnosing corneal and limbal neoplasm.

The statistics of images included in the study are listed in Table 1 consisting of images with eye diseases affecting the corneal photography (four common corneal diseases plus cataract) as well as images of disease free (Normal) eyes. The algorithm was trained with a randomly selected 80% of the retrospective dataset and the remaining 20% of images was used to validate the algorithm. Receiver operating characteristic (ROC) curves were plotted to assess specificity and sensitivity. The areas under the curve (AUC) of the proposed deep learning algorithm were 0.930 (95% confidence interval 0.904–0.952) for infectious keratitis, 0.934 (95% confidence interval 0.911–0.957) for non-infectious keratitis, 0.939 (95% confidence interval 0.910–0.969) for corneal dystrophy or degeneration, and 0.951 (95% confidence interval 0.921–0.986) for corneal and limbal neoplasm, 0.903 (95% confidence interval 0.881–0.924) for cataract, and 0.951 (95% confidence interval 0.929–0.973) for normal ocular surface (Fig. 5a, Supplemental Fig. 2).

We also prospectively tested the algorithm’s performance versus ten board-certified ophthalmologists in identifying corneal diseases from 510 patients newly enrolled in the outpatient clinic of two tertiary eye centers (Table 1). For each test, previously unseen images with a definite diagnosis were displayed and ophthalmologists were asked to determine the disease. As shown in Fig. 5b, each ophthalmologist provided a single diagnosis per image represented by a single blue point on the graph. The red points are the average of all the ophthalmologists for each task (calculated from *n* = 510 and 10 tested ophthalmologists for infectious keratitis, non-infectious keratitis, corneal dystrophy or degeneration, and corneal and limbal neoplasm, respectively). The algorithm achieves superior performance to an ophthalmologist if the sensitivity–specificity point of the ophthalmologist lies below the green curve. The AUC of ROC curves of the algorithm for each corneal disease type was over 0.910 and in general it had sensitivity and specificity similar to or better than the average values of all ophthalmologists (Fig. 5b). Importantly, we observed negligible changes in AUC (all < 0.04, Supplemental Table 1) when we compared the retrospective dataset used to build the algorithm (Fig. 5a) with the independent real-world prospective dataset (Fig. 5b). This suggests that the results of our algorithm are reliable and generalizable across different datasets.

Confusion matrices for the algorithm and two ophthalmologists across the normal, cataract and four common corneal diseases classifications reveal similarities in misclassification between human experts and the algorithm (Fig. 5c). Many images are mistaken as class 1, cataract, owing to the high variability of diseases in this category. Note that non-infectious keratitis is commonly confused for other diseases both by the algorithm and ophthalmologists. The high variability of this disease is challenging to visually diagnose. Similarly, the distribution across row five in all plots shows the difficulty of classifying corneal dystrophy or degeneration, which tends to be diagnosed as infectious keratitis. Examples of misclassifications by the algorithm in the prospective dataset are examined in Fig. 4 with domain expert’s explanations.

In addition, we also compared the performance of our algorithm with four previous reported methods, namely Inception-v3^8^, ResNet^16^, DenseNet^23^ and Ensemble^24^. Using similar approach as Gulshan et al.^10^, instead of training new models from scratch, we applied a fine-tuning strategy directly on pre-trained models of Inception-v3^8^, ResNet^16^ and DenseNet^23^ using a multi-step retraining strategy. In this strategy, we gradually unfroze the layer weights in steps with the first few layers being unfrozen last. In these steps, we also used progressively reduced learning rates from 0.0001 to 0.000001 and with other parameters unchanged. The Ensemble^24^ model combined all backbone features extracted from the other three models (namely the Inception-v3, ResNet, DenseNet models) and applied a tree-based classifier for the final classification. As shown in Fig. 5d, our algorithm outperformed over all four existing methods in identified corneal diseases. For example, for infectious corneal disease, our algorithm achieved AUC 0.960 whereas the Inception-v3^8^, ResNet^16^, DenseNet^23^ and Ensemble^24^ models achieved AUC 0.950, 0.938, 0.954, and 0.908, respectively.

**Figure 6:**
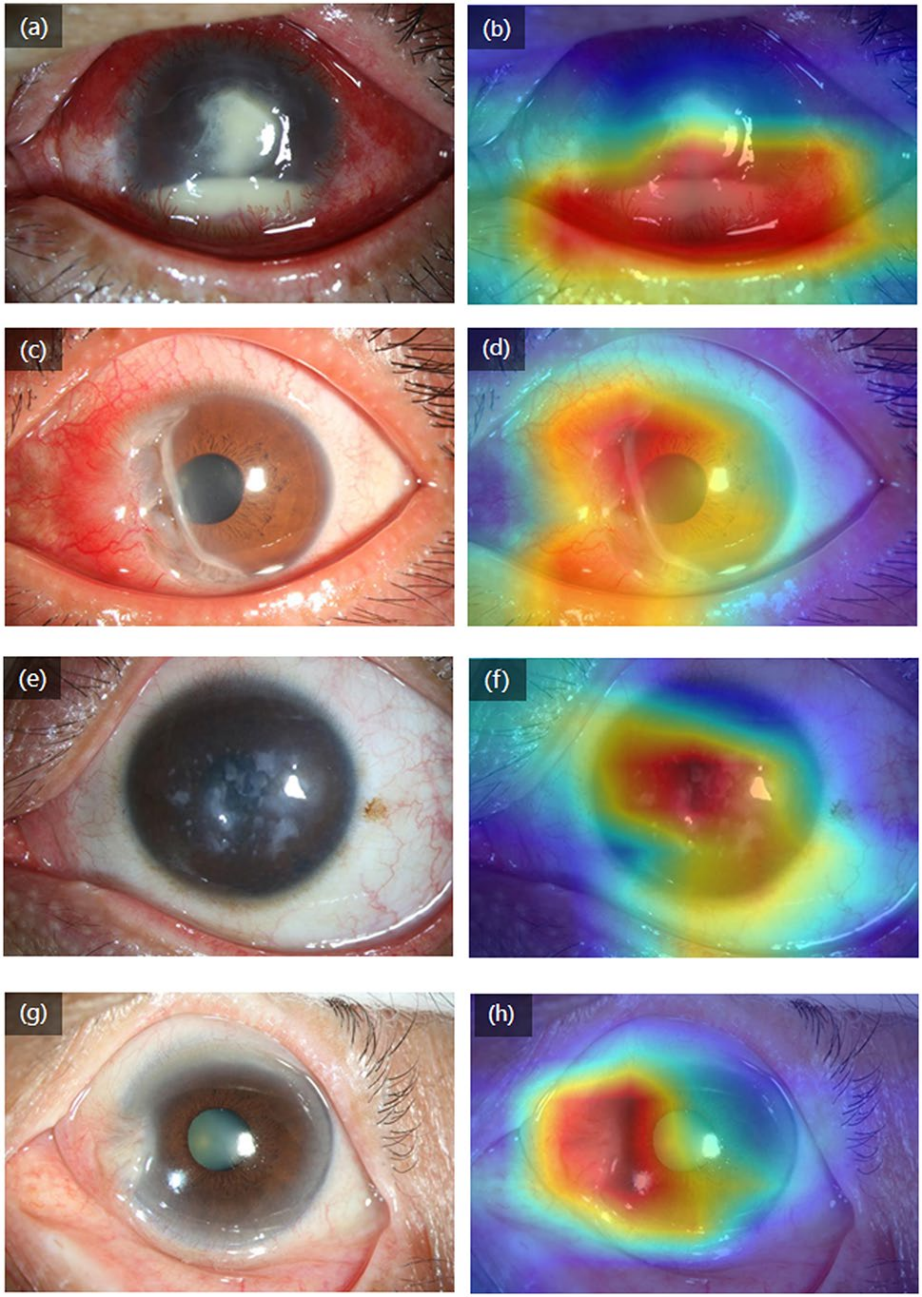
The heatmap for images with various referral corneal diseases. These visualizations are generated automatically, locating regions for closer examination after a patient is seen by a consulting ophthalmologist. The bluer the color, the lower the attention of the model; the redder the color, the higher the attention of the model. (**a)** An ocular surface image shows a case with infectious keratitis. (**b)** The heatmap highlights the pathologic regions in the central and inferior cornea. **(c**,**d)** The heatmap reveals pathologic regions in the nasal cornea of a case with a peripheral corneal ulcer. **(e**,**f)** The heatmap indicates pathologic regions in the whole cornea of a case with macular corneal dystrophy. **(g**,**h)** The heatmap highlights pathologic regions in the nasal quadrant of a case with pterygium.

In an effort to improve efficiency in a clinical setting, we also created a heatmap via gradient-weighted class activation mapping (Grad-CAM) algorithm^25^, which can produce visual explanations for convolutional neural network based deep learning models, thereby establishing prediction trust and interpretation for physicians. In our case, it helped to indicate the potential corneal lesion regions for further examination by physicians (Fig. 6). Grad-CAM uses the gradient information flowing into the last convolutional layer to understand the importance of each neuron for a decision of interest thereby highlighting the important regions in the image for predicting the disease. It first computes the gradient of the score for a given class with respect to feature maps of a convolutional layer. Then, these gradients are averaged-pooled to obtain the neuron importance weights. Finally, the coarse heat-map for a given class is generated via a weighted combination of forward activation maps followed by a ReLU function. As an example, the ocular image in Fig. 6a highlights regions of corneal edema and opacity, as well as hypopyon, in the central and inferior quadrants, indicating infectious keratitis.

## Discussion

Ocular surface examinations are recommended for detecting corneal diseases^26,27^. Furthermore, access to OCT imaging can pose logistical and economic challenges for many patients, particularly normal subjects. For the above reasons, we chose ocular surface images for developing the deep learning algorithm. In order to prevent overfitting problems associated with deep learning algorithms, we split the images into training, validation, and test datasets. The AUCs based on both the validation and test datasets showed that the algorithm is generalizable and can provide accurate results in a real-world setting for cases not previously examined. In addition to classifying the images, the heatmap visualization feature accurately detects abnormal corneal regions in the images, enabling clinical review and the verification of the algorithm’s diagnoses. In a recent published study, Li et al^28^ reported a workflow for the segmentation of anatomical structures and the annotation of pathological features in slit-lamp images to improve the performance of a deep-learning algorithm for diagnosing ophthalmic disorders. By using 1,772 slit-lamp images, they could detect corneal opacity and corneal neovascularization with acceptable sensitivity and specificity. Unlike our study, they tried to detect the clinical signs of the pathological cornea rather than the diagnosis of patients with corneal diseases.

Our deep learning algorithm showed an excellent diagnostic performance for the detection of corneal disease when applied in outpatient clinics and it also strived to differentiate diseases that are easily confused with each other, especially corneal lesions located in the central cornea, particularly in the pupil area, and cataracts. While our algorithm performed well in calling these difficult cases, it must be improved in the future for truly accurate and robust detection. One potential improvement would be to train our algorithm on corneal OCT images, targeting specific characteristics of the corneal sagittal plane.

Several limitations to our study should also be mentioned. First, the collection of a larger image dataset with additional types of ocular surface diseases from different digital slit lamp photography systems and hospitals (not only level 2 corneal disease labels in the Fig. 1a, but also level 3 labels and the other ocular diseases such as glaucoma, uveitis, and conjunctival disease) is warranted as additional gains achieved by increasing the diversity of training data. However, only images covering and centering around the cornea can be applied. Second, we did not perform a detailed analysis for level 3 labels in the current study due to the limitation of our sample size (about 160–200 images for each label in level 3). We will enroll more images to make a comparison with the network that has the finest level3 classifier in the future. Third, there may be an ascertainment bias in the prospective part of our study as we noted that outpatients with decreased best-corrected visual acuity were more likely to participate. This suggests the algorithm may not perform as effectively on images having corneal disease without vision problems. Finally, due to the limited resources, our algorithm has been trained to identify only the ocular surface diseases listed in the methods section. The algorithm may miss other eye diseases with normal ocular surface for which it was not trained to identify. Further research, especially a prospective study focusing on patients’ prognosis undergoing the AI diagnosis-oriented therapy, is necessary to determine whether it could ultimately improve patient care and outcomes as well as save physicians’ time and energy.

In conclusion, based on a large dataset of ocular surface photographs, we developed a deep learning algorithm that has high sensitivity and specificity for detecting four common corneal diseases. In addition, we repeated these results in a prospective study in outpatient clinics. For the proper clinical application of our method, further tests are needed to overcome the variation in images taken by different imaging systems and to optimize our algorithm for different demographics during clinical use. Finally, it should be noted that deep learning algorithms benefit from every additional piece of data they examine. As such, we envision that routine use of this algorithm in a clinical setting will result in continued improvement of diagnoses made.

## Supplementary information

Supplementary Figure 1.

Supplementary Figure 2.

Supplementary Table 1.

## Data Availability

Data and source code are publicly available under the restrictions of scientific research and publication review purpose only. The training and testing data are publicly available at Shanghai EENT Hospital of Fudan University’s Database (https://223.167.111.163:7000/link/F655D03ECAE77327EA99D854833FC6CB).

## Code availability

Source code are available. Project name: Corneal Diseases Classification with Deep Learning. Project home page: https://gigantum.com/guoyouwen/corneal-disease-project. Operating system(s): ubuntu 16.4 64bit. Programming language: Python 3.6. Other requirements: Pytorch 1.0+, cnn_finetune: pip install cnn_finetune. License: GPL.
