# Supplementary figures and images for: Deep learning for identifying corneal diseases from ocular surface slit-lamp photographs
Source: Sci Rep. 2020 Oct 20;10:17851. doi: 10.1038/s41598-020-75027-3 (PMC7576153; doi:10.1038/s41598-020-75027-3)

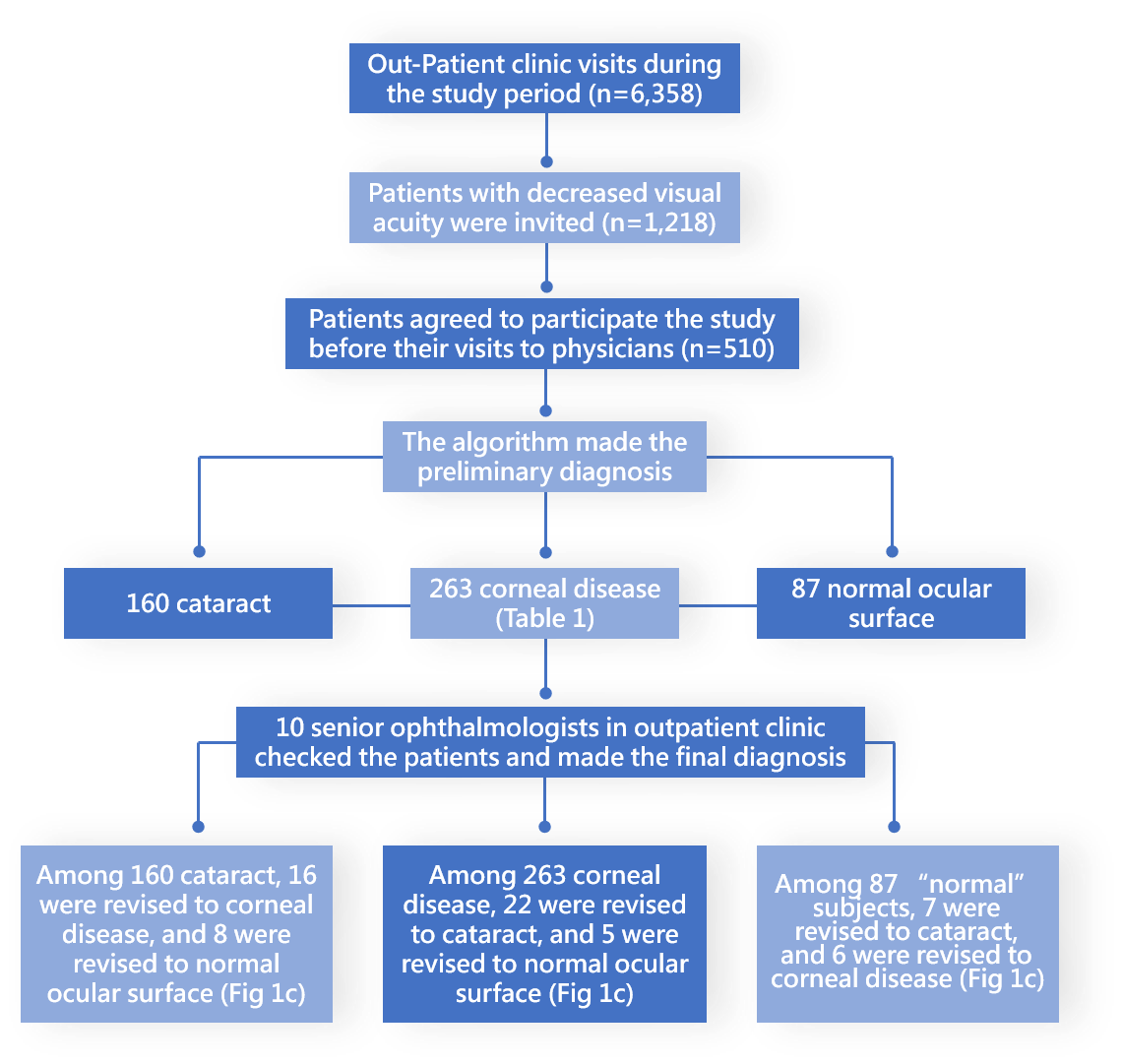

Supplement: Supplementary file 1 — Supplementary Figure 1. [file 41598_2020_75027_MOESM1_ESM.tif]

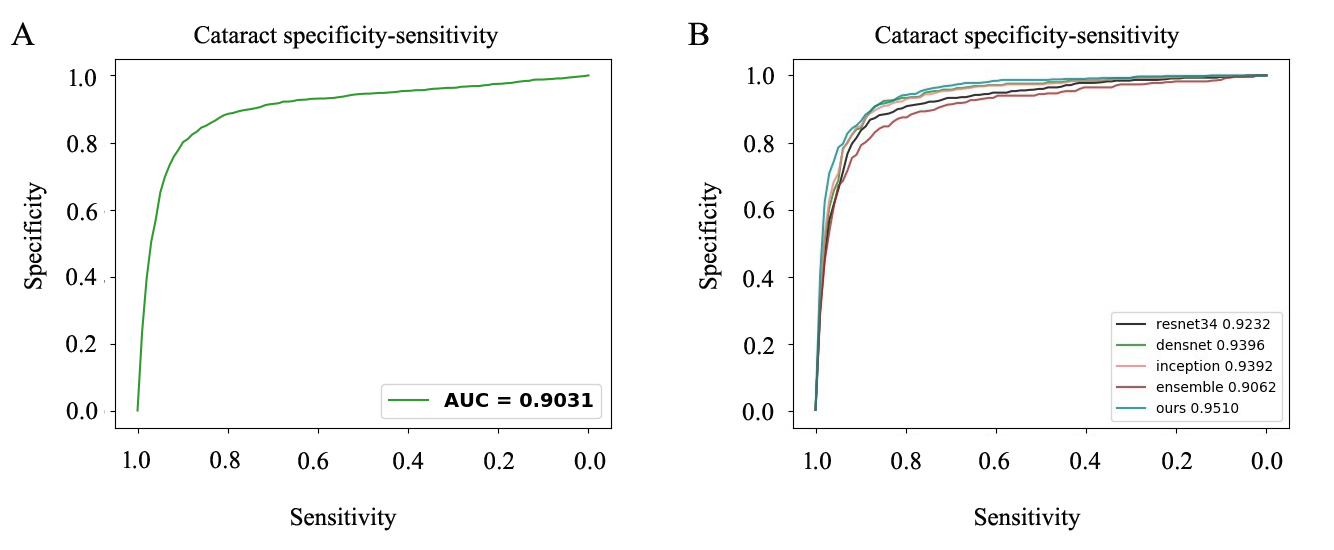

Supplement: Supplementary file 2 — Supplementary Figure 2. [file 41598_2020_75027_MOESM2_ESM.tif]
