# Supplementary material for: Deep learning for identifying corneal diseases from ocular surface slit-lamp photographs
Source: Sci Rep. 2020 Oct 20;10:17851. doi: 10.1038/s41598-020-75027-3 (PMC7576153; doi:10.1038/s41598-020-75027-3)
Supplement: Supplementary file 3 — Supplementary Table 1. [file 41598_2020_75027_MOESM3_ESM.doc]

**Title: Deep Learning for Identifying Corneal Diseases from Ocular Surface Slit-Lamp Photographs**

**Author:** Hao Gu*^1^, Youwen Guo*^2^, Lei Gu*^3,4^, Anji Wei^4^, Shirong Xie^4,5,6^, Zhengqiang Ye^4,5,6^, Jianjiang Xu^4,5,6^, Xingtao Zhou^4,5,6^, Yi Lu^4,5,6^, Xiaoqing Liu^#7^, Jiaxu Hong^#4,5,6^

**Affiliation:**

1 Department of Ophthalmology, The Affiliated Hospital of Guizhou Medical University, Guiyang, China

2 Hisense Medical, Qingdao, China

3 Boston Children Hospital, Harvard Medical School, Boston,USA

4 Department of Ophthalmology and Visual Science, Eye, and ENT Hospital, Shanghai Medical College, Fudan University, 83 Fenyang Road, Shanghai, China

5 Shanghai Key Laboratory of Visual Impairment and Restoration, Science and Technology Commission of Shanghai Municipality, Shanghai, China

6 Key Laboratory of Myopia, Ministry of Health, Shanghai, China

7 Deepwise AI Lab, Beijing,China.

*These authors contributed equally to this paper.

#Correspondence and requests for materials should be addressed to Jiaxu Hong ([jiaxu_hong@163.com](mailto:jiaxu_hong@163.com)) and Xiaoqing Liu, ([xiaoqing.liu@ieee.org](mailto:xiaoqing.liu@ieee.org)).

**Running head:** Artificial intelligence for identifying corneal diseases

**Supplemental Table 1. The Areas Under the Curve (AUC) of the Algorithm in the Two Datasets**

|  | **Retrospective dataset (95%CI)** | **Prospective dataset (95%CI)** |
| --- | --- | --- |
| 1. **Normal Subjects** | 0.951 [0.929 - 0.973] | 0.911 [0.862 - 0.955] |
| 1. **Cataract** | 0.903 [0.881 - 0.924] | 0.870 [0.830 - 0.908] |
| 1. **Infectious keratitis** | 0.930 [0.904 - 0.952] | 0.960 [0.946 - 0.973] |
| 1. **Non-infectious keratitis** | 0.934 [0.911 - 0.957] | 0.892 [0.848 - 0.931] |
| 1. **Corneal dystrophy or degeneration** | 0.939 [0.910 - 0.969] | 0.895 [0.821 - 0.956] |
| 1. **Corneal Neoplasm** | 0.951 [0.921 - 0.986] | 0.957 [0.939 - 0.975] |
| *In the current study, corneal diseases include infectious keratitis, non-infectious keratitis, corneal dystrophy or degeneration, and ocular surface neoplasm affecting the cornea. | | |
